# Engineering Smart Scaffolds for Osteomyelitis: Integrating Nanotechnology, Drug Delivery, and Tissue Regeneration—A Narrative Review

**DOI:** 10.3390/jfb17080379

**Published:** 2026-08-03

**Authors:** Chayse Baker, Caleb Jolley, Ian Alexander, Elijah Lee, Hemalatha Kanniyappan, Aftab Merchant

**Affiliations:** 1 Kirk Kerkorian School of Medicine, University of Nevada, Las Vegas, NV 89106, USA; bakerc20@unlv.nevada.edu (C.B.); jollec2@unlv.nevada.edu (C.J.); alexai1@unlv.nevada.edu (I.A.); leee72@unlv.nevada.edu (E.L.); 2 Department of Biomedical Engineering, Riccio College of Engineering, University of Massachusetts Amherst, Amherst, MA 01003, USA

**Keywords:** osteomyelitis, biomaterial scaffolds, bone tissue engineering, targeted drug delivery, bone regeneration, smart scaffolds

## Abstract

Osteomyelitis is a severe, progressive bone infection affecting approximately 21.8 per 100,000 individuals in the United States and remains a major challenge in orthopedic and reconstructive medicine. *Staphylococcus aureus* (*S. aureus*), responsible for nearly 75% of cases, is the most common causative pathogen. Current gold-standard management involves culture-directed antibiotic therapy administered for 4–6 weeks, often combined with surgical debridement and the use of antibiotic-loaded cement spacers. Despite these interventions, treatment frequently fails to achieve complete infection eradication, with recurrent or persistent disease reported in up to 40% of cases, contributing to chronic inflammation, impaired bone healing, and long-term functional deficits. These limitations highlight the need for therapeutic strategies that address infection control and bone regeneration. A narrative literature review was conducted to evaluate emerging tissue-engineered scaffold-based biomaterials as potential alternatives to conventional treatment. Emerging evidence suggests antimicrobial-loaded matrices, bioactive scaffolds, and stimuli-responsive biomaterials can provide localized drug delivery, structural support, and enhanced osteogenesis while improving infection control and osteointegration. Advances in targeted drug delivery, immunomodulatory biomaterials, and computational scaffold design further suggest opportunities for more effective therapies. These findings highlight the potential of scaffold-based biomaterials to improve osteomyelitis management, although further clinical studies are required.

## 1. Introduction

Osteomyelitis is a complex inflammatory infection of bone tissue, caused by microorganisms that can lead to progressive bone destruction, and, in severe cases, necrosis. It affects approximately 21.8 per 100,000 people annually in the United States, with higher incidence observed in men, older adults, and individuals with diabetes [1]. *Staphylococcus aureus* (*S. aureus*) is the most common causative pathogen, accounting for approximately 75% of osteomyelitis cases [2], though other pathogens—including coagulase-negative staphylococci, streptococci, and *Escherichia coli* (*E. coli*)—also contribute. In specific populations, organisms such as *Salmonella* (notably in sickle cell disease), *Pseudomonas* (in injection drug users), and polymicrobial infections in diabetic foot osteomyelitis are frequently implicated [3].

Osteomyelitis continues to pose a significant challenge in orthopedic and reconstructive medicine because recurrent or persistent osteomyelitis occurs in up to 40% of cases [4]. Chronic osteomyelitis has been associated with a 1.94-fold increased risk of fractures compared with control groups. It may also lead to bone defects and limb shortening due to processes such as surgical debridement, osteonecrosis, osteolysis, and sequestrum formation [5,6]. In some cases, the infection may extend into surrounding soft tissues, necessitating more aggressive interventions such as en bloc resection to fully eradicate the infection [7]. Additionally, bacteremia arising from osteoarticular infections accounts for approximately 1.8% of all bacteremia’s, with 40% attributed to vertebral osteomyelitis and 17% to peripheral osteomyelitis [8]. Chronic osteomyelitis has also been suggested to have a potential association with squamous cell carcinoma, although further research with longer follow-up periods is needed to confirm this relationship [9]. While prolonged antibiotic therapy remains a cornerstone of management, its typical six-week duration raises concerns regarding the potential development of antibiotic resistance [10].

Early treatment of acute osteomyelitis is associated with favorable outcomes, whereas delayed or inadequate therapy can lead to complications such as recurrent or persistent osteomyelitis, pathologic fractures, bone deformities, systemic infection, and involvement of adjacent soft tissues [11,12]. Although no universal timeline clearly distinguishes early from delayed treatment, one study of acute osteomyelitis in children found that hospital admission and initiation of antibiotics within five days of symptom onset were associated with improved recovery and outcomes [13].

The current gold standard treatment for osteomyelitis involves culture-directed antibiotic therapy, typically administered for 4–6 weeks, and can be combined with surgical debridement and/or cement spacers when necessary. Treatment strategies are largely influenced by antibiotic penetration into bone and the Cierny–Mader classification system [14,15]. In acute osteomyelitis, management often consists of culture-directed antibiotic therapy alone, as vascular supply to the affected region is generally preserved, allowing adequate antibiotic penetration. In contrast, chronic osteomyelitis is commonly guided by the Cierny–Mader classification system, which considers both the extent of bone involvement and host physiological status [15]. In these cases, necrotic bone, abscess formation, and bacterial biofilm are frequently present, impairing antibiotic penetration and necessitating surgical debridement of infected tissue. For patients requiring debridement and management of resulting bone defects, a two-stage approach known as the Masquelet technique is often used. The first stage involves debridement, drainage, removal of necrotic bone tissue, and insertion of an antibiotic loaded polymethyl methacrylate (PMMA) cement spacer into the resulting dead space. The second stage requires the removal of the spacer within 4–8 weeks of placement followed by cancellous bone grafting [16,17].

However, the Masquelet technique has its limitations. Its primary drawback is that PMMA, commonly known as bone cement, is an inert material that fills bone defects and stabilizes implants or prosthetics without supporting new bone formation. In contrast to tissue-engineered scaffolds, which actively promote bone regeneration and cellular growth, PMMA functions only as a temporary mechanical interlock that secures the implant in place [18]. In the two-stage approach, surgical removal of the PMMA spacer during the second stage introduces additional risks, as the cement may become a site of bacterial colonization once its antibiotic release declines or ceases entirely [16]. A systematic review and meta-analysis reported that the most common complications associated with this technique were superficial and deep surgical site infections, occurring in 4.9% and 4.4% of patients, respectively. Other complications include failure of one of the stages, resulting in persistent infection or nonunion of bone regeneration, affecting up to 18% of patients [19]. Additionally, up to 26.7% of patients required further surgical interventions. PMMA spacers can also reach temperatures as high as 124 °C, potentially causing thermal necrosis to adjacent tissues, compromising local vascularization, and disrupting membrane formation at the bone cement interfaces [20].

Previous reviews have examined individual aspects of osteomyelitis management, including antibiotic therapies and isolated biomaterial strategies; however, these approaches are typically evaluated in isolation and do not reflect the multifactorial nature of the disease [10,21,22]. Effective treatment requires simultaneous infection control and bone regeneration, objectives that remain inadequately addressed by conventional or single-modality approaches [15,22,23].

This narrative review addresses this gap by integrating antimicrobial, immunomodulatory, stimuli-responsive, and computationally optimized scaffold strategies within a unified framework. By synthesizing these emerging scaffold-based strategies relevant to osteomyelitis management—including approaches that address infection control, localized drug delivery, bone regeneration, immunomodulation, and personalized scaffold design—this review highlights multifunctional scaffold systems with the potential to improve therapeutic outcomes and inform future translational research in osteomyelitis. Because many of these emerging technologies have not yet been evaluated directly in osteomyelitis models, this review distinguishes osteomyelitis-specific evidence from studies conducted in implant-associated infection prevention, antimicrobial delivery, bone regeneration, and related preclinical contexts, emphasizing their translational potential rather than presenting them as established clinical therapies for osteomyelitis.

## 2. Literature Search Strategy

A narrative literature review was conducted using PubMed, UNLV Library Search, OpenEvidence, and Elicit to identify relevant studies on osteomyelitis and scaffold-based biomaterials for bone regeneration. Articles published between 1997 and 2026 were considered, with emphasis on preclinical, translational, and emerging clinical studies evaluating antimicrobial, bioactive, and tissue-engineered scaffold strategies.

Studies were included if they focused on biomaterial scaffolds for bone repair, bone tissue engineering, scaffold-based therapeutic strategies, or emerging technologies with potential applications in osteomyelitis management, including antimicrobial delivery systems, immunomodulatory approaches, and regenerative therapies. Studies unrelated to these topics or involving non-relevant disease processes were excluded. Titles and abstracts were screened for relevance, followed by a full-text review of selected articles.

Search terms included “osteomyelitis,” “bone scaffolds,” “biomaterials,” “antimicrobial delivery,” and “tissue regeneration.” Reference lists of included articles were also reviewed to identify additional relevant studies.

This study was conducted as a narrative review and did not follow PRISMA guidelines; however, efforts were made to ensure a comprehensive and balanced selection of relevant literature and to distinguish direct osteomyelitis evidence from studies with potential translational relevance.

## 3. Results

### 3.1. Biomaterial-Based Scaffold Strategies for Osteomyelitis Treatment

To address the limitations of conventional treatments, tissue-engineered biomaterial-based scaffolds are being implemented to further encourage tissue regeneration and represent a promising advancement in osteomyelitis management. In this review, tissue-engineered scaffolds are defined as porous biomaterials that are three-dimensional structures, mimicking the extracellular matrix of bone tissue and promoting bone tissue repair and regeneration [24]. Such fabrication techniques offer significant advances over conventional osteomyelitis treatments by simultaneously preventing biofilm formation and regenerating lost bone tissue. These 3D scaffolds can deliver antibiotics with tailored kinetics and incorporate antimicrobial ions, providing sustained therapeutic efficacy and reducing the risk of bacterial resistance [25,26,27,28].

**Figure 1:**
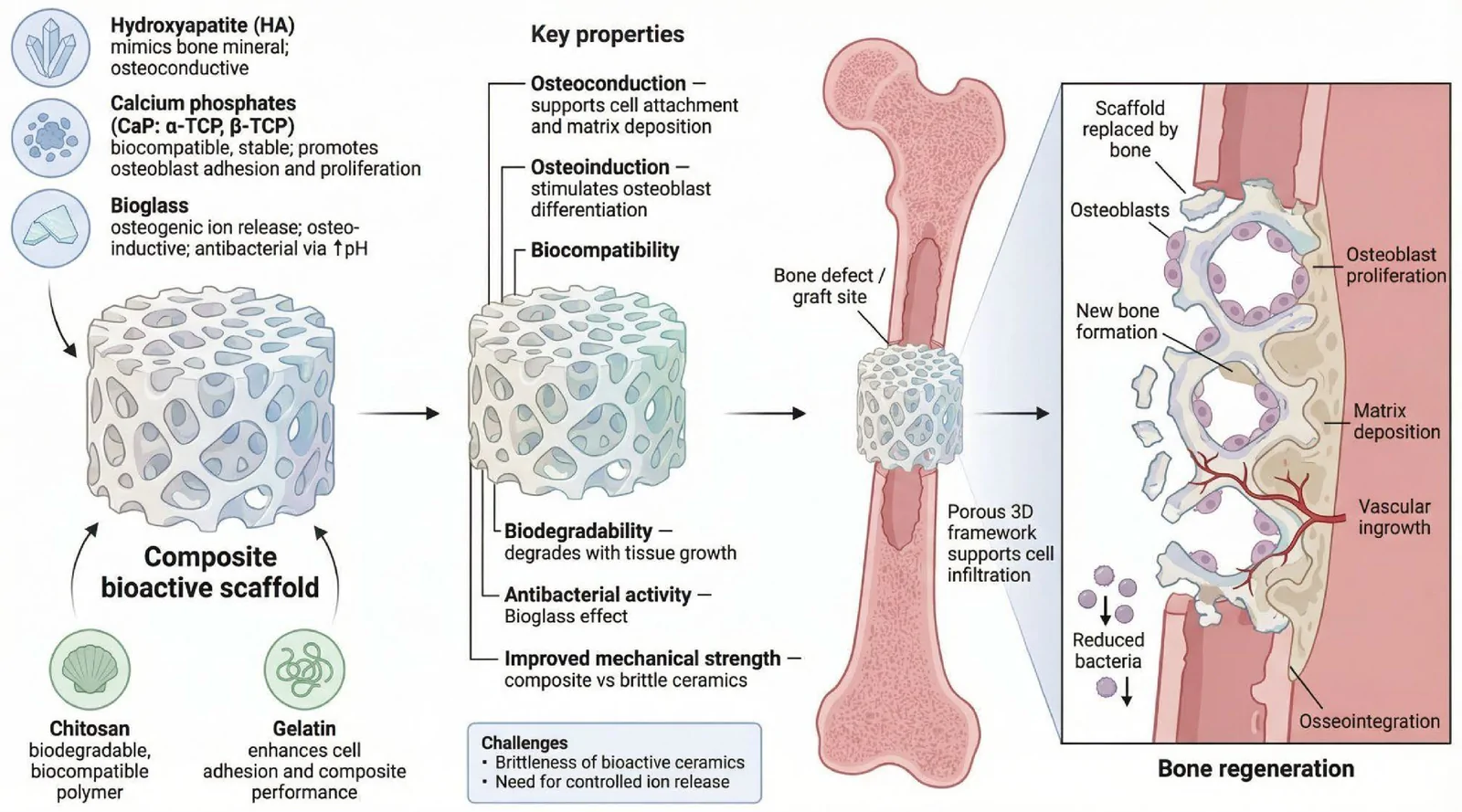
**Bioactive, osteoconductive, and synthetic scaffolds for bone regeneration.** Composite synthetic scaffolds support bone defect repair by combining structural support with biologic activity. Hydroxyapatite (HA), calcium phosphates, and Bioglass promote osteoconduction, osteoinduction, and antibacterial activity, while polymeric components such as chitosan and gelatin improve scaffold biocompatibility, biodegradability, and mechanical performance. Created in BioRender. Alexander, I. (2026) https://BioRender.com/xo1rlek (Accessed on 20 July 2026).

Ghassemi et al. describe bone scaffolds as biodegradable implants that promote osteoblast attachment and proliferation following osseous injury. Biomaterials including composites, metals, ceramics, and polymers possess varying degrees of osteoconductive and osteoinductive potential. Osteoconductive properties of scaffolds provide a framework that guides the migration of bone-regenerating cells—including mesenchymal stem cells, osteoblasts, and osteoclasts—into the interior of the scaffold. In contrast, osteoinductive properties actively promote the differentiation and maturation of osteogenic cells, stimulating new bone formation (Figure 1). While osteoconduction and osteoinduction describe scaffold-mediated support and stimulation of bone-forming cells, osteogenesis refers specifically to new bone formation driven by viable osteogenic cells themselves. Thus, scaffolds are inherently osteoconductive and may be osteoinductive, whereas true osteogenesis requires the presence and activity of viable bone-forming cells rather than material alone [29].

### 3.2. Antimicrobial-Loaded Scaffolds

Scaffolds support osteogenic cell attachment and proliferation; however, implanted biomaterials may increase the risk of secondary infection when antimicrobial protection is inadequate [30]. Scaffold sterilization may be used to mitigate this risk; however, challenges with sterilization also exist. The core problem is that various sterilization methods are known to cause scaffold deterioration. For example, one group of researchers found that sterilizing PLGA scaffolds with ethylene oxide caused the scaffold to shrink to 60% of its initial volume while gamma-irradiation caused loss in molecular weight [31]. Other options to address secondary infection risk include coating scaffolds with antibiotics before placement so an aseptic environment is established. Combining scaffold-based delivery with multiple antimicrobial agents may further improve treatment of complex bone infections. Antimicrobial-loaded scaffolds hold great potential, but their effectiveness is limited by the difficulty of fully removing all infected tissue during surgery. Residual bacteria that remain can evade local treatment and may also develop resistance, leading to persistent or recurrent infection [28,32,33,34].

**Figure 2:**
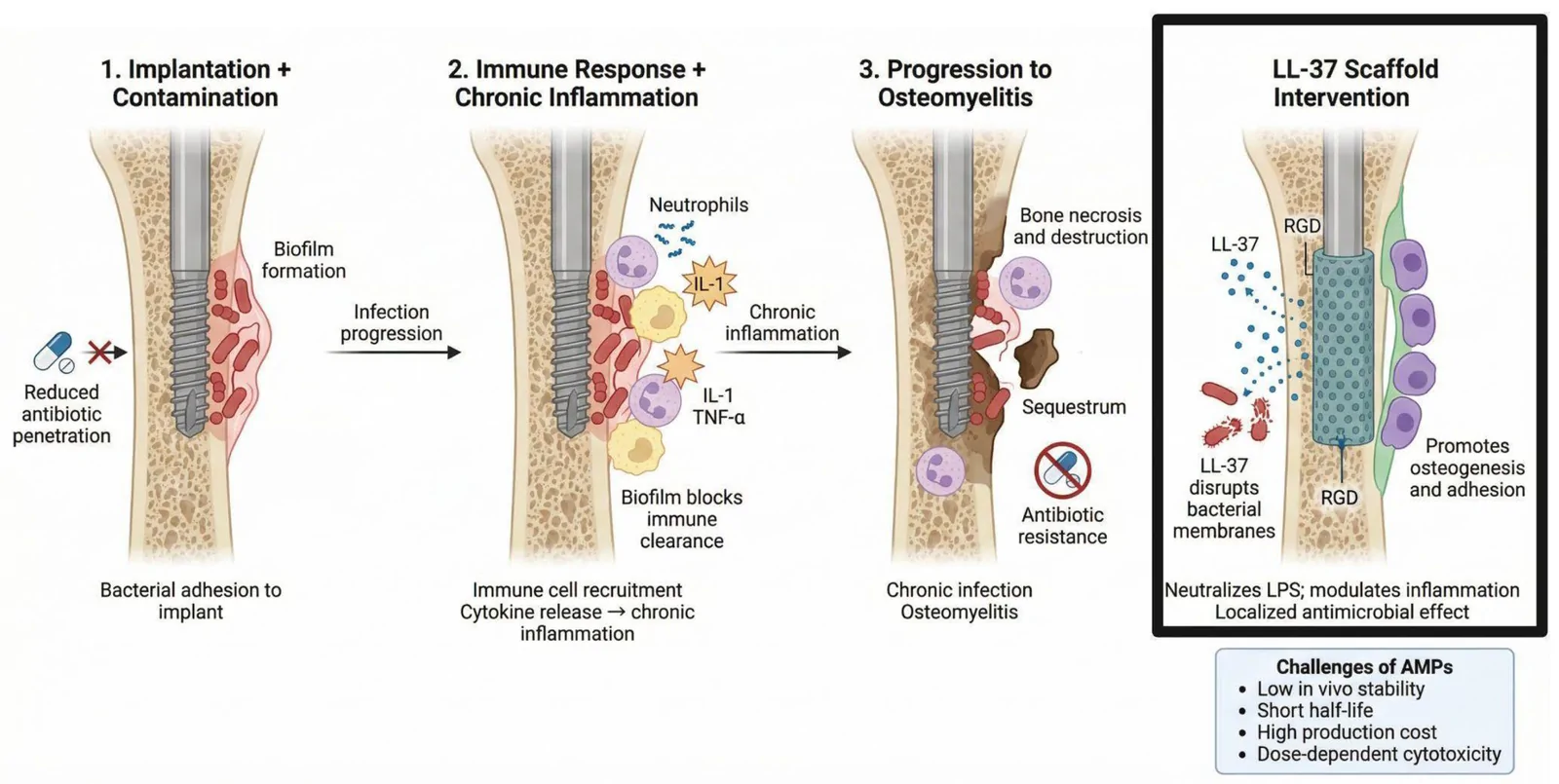
**Pathophysiology of implant-associated osteomyelitis and therapeutic role of LL-37-infused scaffolds.** Implantation of foreign bodies introduces the possibility of bacterial contamination. Subsequent biofilm formation impairs the host immune response and limits bacterial clearance. LL-37-infused scaffolds provide localized antimicrobial activity by disrupting bacterial membranes, neutralizing lipopolysaccharide (LPS), modulating inflammation, and enhancing osteogenesis. Color coding is as follows: bone (tan), implant hardware (gray), bacteria and biofilm (red), and pro-inflammatory cytokines including IL-1 and TNF-α (orange stars); recruited immune cells such as macrophages and other leukocytes are shown in purple and yellow, and necrotic bone and sequestrum in brown. In the intervention panel, the LL-37 peptide is depicted as blue dots, the biodegradable scaffold in teal, and host osteogenic cells in purple. Created in BioRender. Alexander, I. (2026) https://BioRender.com/0d4t4q1 (Accessed on 20 July 2026).

Increasing antibiotic resistance poses a challenge when bacterial infections occur following implantation procedures (Figure 2). One promising strategy to reduce reliance on systemic antibiotics is the use of antimicrobial peptide (AMP)-infused scaffolds. Naturally found in human neutrophils, LL-37 can be combined with AMP-infused scaffolds [35]. LL-37 disrupts bacterial membranes, neutralizes lipopolysaccharide (LPS) toxicity, and modulates cytokine release and immune cell differentiation while exhibiting limited toxicity toward host cells. It demonstrates antibacterial activity against bacteria such as *Pseudomonas aeruginosa*, *E. coli*, and *S. aureus*. Notably, it is less effective against Methicillin-resistant *S. aureus* (MRSA), *Proteus mirabilis*, and *Candida albicans* in high salt (100 mM) concentrations [36]. Beyond direct antibacterial activity, AMP-infused scaffolds can also promote tissue regeneration by being combined with other peptides containing RGD (arginine-glycine-aspartic acid) motifs to increase cellular adhesion and LL-37 to increase osteogenic activity [37,38]. Clinical translation of AMP-based scaffolds remains limited by several important challenges. Their susceptibility to proteolytic stability degradation shortens in vivo half-life, dose-dependent cytotoxicity restricts therapeutic dosing, and high manufacturing costs continue to limit widespread application [39,40]. Although advances in peptide engineering and scaffold design are beginning to address these barriers, improving stability while maintaining antimicrobial efficacy remains a major focus on ongoing research.

Implantation devices, such as fracture fixation hardware (plates, screws, intramedullary nails), joint prostheses, and bone graft substitutes, increase the risk of microbial colonization and subsequent osteomyelitis, particularly by MRSA and other microbial pathogens. Given these risks, there is growing interest in incorporating antimicrobial strategies directly into implantable orthopedic and reconstructive materials. To reduce the risk of postoperative infections and osteomyelitis, biodegradable scaffolds can be engineered to release silver ions in a controlled manner. This localized delivery provides broad-spectrum bactericidal action that delays bacterial growth and prevents biofilm formation while simultaneously supporting stem cell osteogenesis and bone mineralization without causing systemic toxicity. Mohiti-Asli et al. demonstrated that scaffolds containing silver decrease the rate of MRSA growth, which reduces the chance for osteomyelitis development [41]. While the precise antimicrobial mechanism is not fully understood, these scaffolds appear to disrupt microbial DNA replication and enzymatic activity while also inhibiting biofilm formation. Importantly, these antimicrobial effects occur without compromising osteoblast proliferation or decreasing the viability of mesenchymal stem cells. The clinical utility of silver-containing scaffolds, however, depends on balancing antimicrobial efficacy with host safety. A proposed therapeutic window of 10–33 μM may minimize cytotoxicity, though evidence remains mixed [42,43]. Although three-dimensional scaffold architecture may further reduce cellular silver exposure, prolonged accumulation of metallic ions remains a concern because of their potential effects on oxidative stress, renal and hepatic function, and immune regulation. Establishing safe and effective dosing strategies will therefore be essential for successful clinical translation [44,45].

### 3.3. Bioactive, Osteoconductive, and Osteoinductive Scaffolds

Soleymani et al. demonstrated that hydroxyapatite (HA)-based scaffolds serve as synthetic osteoconductive scaffolds, mimicking the structure and function of calcified tissues such as teeth and bone [27]. HA is commonly used as a coating material to strengthen bone implants and enhance targeted antibacterial therapy [46]. Beyond coatings, HA can also be incorporated into implant materials to further improve their structural and biological performance. Despite these advantages, the inherent brittleness of HA limits its use as a standalone implant material because of its susceptibility to mechanical damage. Rather than abandoning HA altogether, there has been a recent focus on developing composite scaffolds that combine HA with complementary biomaterials to overcome these limitations while enhancing mechanical strength, antibacterial activity, and osseointegration [27,47,48].

Bioglass and calcium phosphate (CaP) scaffolds provide many benefits for bone healing. The most common CaPs include HA, alpha-tricalcium phosphate (ɑ-TCP), and beta-tricalcium phosphate (ꞵ-TCP) [49]. They are promising biomaterial candidates due to their similarity to the inorganic phase of bone, which is composed of approximately 70% calcium phosphate [50,51]. CaPs form direct bonds with bones and are known to increase adhesion, proliferation, and differentiation of osteogenic cells [50]. In addition to being more biocompatible than other ceramics or inorganic nanoparticle categories, CaPs are also favored for their stability, safety, and low-cost [51]. Bioglass is another bioactive ceramic that interacts with bone and its surrounding biological environment to promote bone growth and regeneration rather than merely serving as an inert placeholder material and is known for its high bioactivity and antimicrobial activity against certain bacteria [49]. Bioglass exhibits osteoinductive behavior and releases ionic dissolution products that stimulate osteoblastic gene expression, thereby enhancing bone regeneration [50]. Consequently, Bioglass has greater potential to stimulate bone regeneration compared to other bioactive ceramics [51]. Bioglass also exhibits antibacterial activity, as its ionic dissociation raises the local pH, disrupting various bacterial cellular functions [49,51,52]. When CaPs such as HA and Bioglass are combined, the resulting scaffold exhibits greater bonding strength, improved mechanical properties, and enhanced bioactivity compared to using either material alone (Figure 1) [51]. Combining them also imparts the antibacterial properties that CaP scaffolds alone lack [49]. Despite these favorable properties, optimizing the performance of Bioglass and CaP scaffolds remains an important engineering challenge. Maintaining controlled ion release is critical for preserving the bioactivity and therapeutic efficacy of Bioglass while avoiding undesirable alterations to the local microenvironment [50]. Furthermore, bioactive ceramics such as CaPs and Bioglass are relatively brittle compared to scaffolds made from synthetic bioresorbable polymers, highlighting the continued need for composite scaffold designs that balance mechanical strength with biological performance [52].

Autografts are the gold standard when treating bone injuries requiring a graft. Although bone possesses intrinsic regenerative capabilities, extensive defects can often exceed its natural healing ability. In such cases, the limited availability of harvestable donor bone makes autografting an insufficient method for large defect repair. If autografts are not feasible, the use of polymeric scaffolds represents a viable alternative [53]. Polymeric scaffolds include materials that are biocompatible, bioactive, biodegradable, non-toxic, and possess both osteoinductive and osteoconductive activity [54]. The scaffolds provide sites for cell attachment and provide mechanical stability, and they stimulate osteoblast proliferation while degrading at a similar rate during new tissue formation [55,56]. Chitosan-based scaffolds satisfy the key features of polymeric scaffolds; however, they are biomechanically inferior to other materials due to their limited capacity to support load-bearing bone repairs [56]. Rather than limiting the use of chitosan, this mechanical weakness has driven the development of composite scaffolds that combine chitosan with complementary polymers, such as gelatin [57]. By integrating the favorable biological properties of chitosan with the mechanical advantages of additional polymers, these composite scaffolds provide a more balanced platform for bone regeneration.

### 3.4. Smart and Responsive Scaffolds

Gao et al. described pH-sensitive hydrogels as polymeric network systems that use chemical bonds and interactions to create environments to respond to appropriate chemical or physical responses such as edema, degradation, contraction, or ion exchange [58]. These dynamic properties have expanded the potential applications of pH-responsive hydrogels in bone and neural tissue regeneration, particularly by enabling targeted drug delivery and regenerating medicines [59,60]. Despite their versatility, the clinical performance of pH-responsive hydrogels ultimately depends on their ability to respond rapidly and predictably within the local tissue microenvironment. Delayed or insufficient responsiveness at target tissue sites may limit therapeutic efficacy, highlighting the need to further optimize stimulus sensitivity before widespread clinical implementation [58,61].

Stimuli-responsive scaffolds represent an innovative technology used to deliver drugs to precise locations in the body [62,63,64]. These systems utilize 3D elastic networks called hydrogels that can be designed to react to specific stimuli such as pH, redox potential, acoustics, temperature, magnetic fields, and light [62,63,64]. Hydrogels promote targeted drug delivery following a predetermined stimulus due to their hydrophilic nature, swelling, and mechanical properties [62]. Hydrogels can be advantageous due to their transport properties, which arise from their high water content and porous three-dimensional structure, thereby protecting encapsulated drugs by shielding them from the surrounding environment. Beyond their transport capabilities, hydrogels are also advantageous because they are biodegradable [65]. This approach may also minimize side effects associated with higher drug doses, as it allows for a reduced frequency and overall quantity of drug administration [62]. Temperature-sensitive hydrogels can be used to respond to localized changes in temperature associated with fever, local infections, or diseases [66]. Similarly, pH-responsive hydrogels work when pH changes cause alterations in polymer chain reactions through the cleavage of acid-labile linkages [62,66]. Although stimuli-responsive scaffolds offer considerable therapeutic potential, their successful clinical translation depends on overcoming several engineering and biological challenges. These include biosafety, mechanical strength, drug loading, and handling concerns [62,65,67]. Furthermore, robust clinical evidence remains limited, underscoring the need for additional clinical studies to establish their long-term safety and therapeutic efficacy [62]. By enabling controlled, site-specific drug delivery, stimuli-responsive scaffolds have the potential to improve the precision of osteomyelitis management as these technologies continue to evolve.

### 3.5. Advanced Antimicrobial Strategies in and Adjacent to Scaffolds

Although the strategy described below is not itself a scaffold-based model, it is reviewed here because Clustered Regularly Interspaced Short Palindromic Repeats (CRISPR)-Cas9 gene editing represents an emerging antimicrobial approach that is increasingly being explored for future incorporation into the scaffold-based delivery platforms, informing the design of the next generation scaffold-integrated antimicrobial strategies.

**Figure 3:**
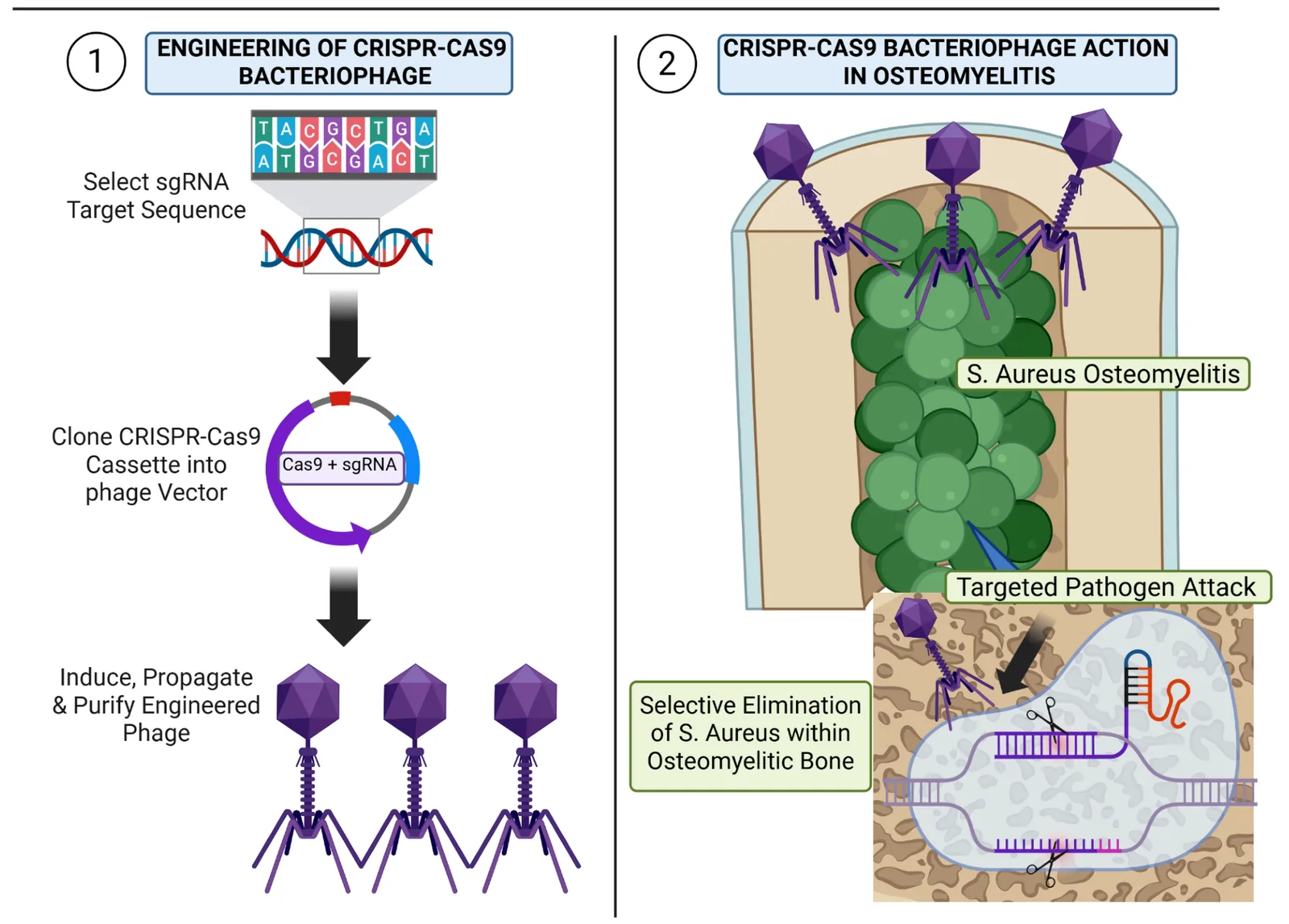
**Engineering and mechanism of CRISPR-CAS9 bacteriophage for targeted treatment of *Staphylococcus aureus* osteomyelitis** [1]. An overview of the engineering process used to develop a CRISPR-Cas9 bacteriophage prior to implantation is shown [2]. Following implantation, the engineered bacteriophage delivers the CRISPR-Cas9 system to *S. aureus*, where targeted DNA cleavage results in selective bacterial elimination. Created in BioRender. Alexander, I. (2026) https://BioRender.com/dnw8we5 (Accessed on 20 July 2026).

Cobb et al. hypothesized that equipping CRISPR-Cas9 with bactericidal capabilities could provide targeted control of *S. aureus* osteomyelitis. The study evaluated bacteriophage-mediated CRISPR-Cas9 delivery rather than a scaffold-based delivery platform; its relevance to this review lies in its potential future incorporation into localized biomaterial systems. To test this, the study developed an in vivo rat model of *S. aureus*-induced osteomyelitis and engineered a bacteriophage carrying CRISPR-Cas9 to selectively attack the pathogen. CRISPR-Cas9 functions through a programmable RNA-guided mechanism in which the Cas9 nuclease forms a complex with a single guide RNA (sgRNA) that recognizes a complementary DNA sequence in the bacterial chromosome. Once bound, Cas9 introduces a double-stranded break at the target site, resulting in chromosomal DNA cleavage and bacterial death. This sequence-specific targeting allows precise elimination of *S. aureus* without disrupting surrounding microbiota, aligning with the engineered bacteriophage mechanism shown in Figure 3 [68]. The modified phage was induced, propagated, purified, and administered without additional preparations beyond the CRISPR implementation, differing from the current gold standard of debridement followed by long-term antibiotic therapy. Although the engineered bacteriophage did not completely eradicate the underlying bone infection, it demonstrated promising efficacy in resolving the associated soft tissue infection, a frequent complication of osteomyelitis [69]. These findings highlight the potential of CRISPR-Cas9 as a highly targeted antimicrobial strategy while also emphasizing that additional optimization is needed before it can replace conventional treatment approaches. Furthermore, other research has demonstrated that this method may be effective for decolonizing antibiotic-resistant bacterial strains, such as vancomycin-resistant staphylococci, or restoring bacterial susceptibility to antibiotics [70,71]. Rather than serving as a standalone therapy, evidence suggests that CRISPR-based approaches may be effective when integrated with surgical interventions to maximize therapeutic efficacy and improve clinical outcomes.

Biofilms create many challenges when treating bacterial infections such as osteomyelitis as they promote antibiotic resistance. Biofilms are highly prevalent and cause more than 60% of bacterial infections [72]. Their formation depends on a bacterial communication process called Quorum Signaling (QS), which coordinates group behavior required for biofilm development. QS is also known to affect signaling pathways in the human body, including G-protein-coupled receptors, toll-like receptors, and immune cell regulation [73,74]. Consequently, targeting QS has emerged as a promising strategy for disrupting biofilm formation and enhancing bacterial susceptibility to conventional antibiotic therapy. The three primary targets include disruption of auto-inducing signaling molecules, QS receptors, and signaling cascades. Interfering with any of these pathways impairs bacterial communication, ultimately preventing the coordinated gene expression necessary for biofilm formation [75,76]. Despite their therapeutic potential, Quorum Sensing-inhibiting scaffolds face several challenges that limit clinical translation. The mechanisms for many QS-inhibiting agents remain unclear, while concerns regarding agent stability, cytotoxicity, and unintended effects on beneficial surrounding bacteria must be addressed before these strategies can be widely implemented [77].

*S. aureus*, including MRSA, is the most common etiologic agent in osteomyelitis. The continued emergence of antimicrobial resistance, including reduced susceptibility to agents such as vancomycin, underscores the need for innovative therapeutic strategies that integrate antibiotics and metal ions into hybrid antimicrobial platforms [78]. Among these hybrid antimicrobial strategies are AMPs, which are key components of the innate immune system that have emerged as promising therapeutic agents in the setting of rising antibiotic resistance [79]. Beyond AMPs, the metal ions used combined with antibiotics such as vancomycin have demonstrated notable efficacy, as they can disrupt bacterial structures and modulate antibiotic uptake via endocytosis, thereby enabling the antibiotic to exert its effects against otherwise resistant bacterial strains [80]. Despite these encouraging findings, the clinical translation of hybrid antimicrobial strategies remains limited by several important challenges. Short in vivo half-lives, together with an incomplete understanding of their toxicity, long-term stability, and therapeutic efficacy, continue to impede their widespread implementation [79].

Nanoparticles (NPs) are a crucial element to delivering bioactive molecules, growth factors, and scaffold-associated therapeutics [81]. NPs facilitate coordination between osteogenic and immune responses, making them promising platforms for promoting bone healing and preventing osteomyelitis [82]. The composition of NPs varies widely and can include materials such as mesoporous silica (MSN), copper, and silver [83,84]. MSNs are particularly advantageous due to their biocompatibility, low cytotoxicity, large surface area, and bioactive surfaces, which together support cellular adhesion, proliferation, and differentiation [83]. These favorable properties also help overcome many of the drug delivery and cytotoxicity limitations associated with other NP systems. For example, some NPs, such as poly(lactic-co-glycolic) acid (PLGA), are synthetic polymers that are inherently hydrophobic, which can impair the loading and delivery of hydrophobic drugs and molecules. Furthermore, synthetic polymers like PLGA generate acidic degradation products that may denature proteins and induce an inflammatory response [84]. Metal-based nanoparticles, such as those containing silver, illustrate a different translational challenge because silver is known to be cytotoxic at high concentrations. Consequently, scaffolds that incorporate silver may pose a risk to eukaryotic cells, as intracellular silver accumulation may disrupt critical cellular processes and compromise membrane integrity [85]. Although nanoparticle-based scaffold systems offer considerable therapeutic potential, their successful clinical implementation depends on balancing enhanced drug delivery with acceptable biocompatibility and safety. Optimizing nanoparticle composition while minimizing cytotoxicity and improving delivery efficiency therefore remains a critical focus on ongoing research.

### 3.6. Immunomodulatory Scaffolds for Infection Control

**Figure 4:**
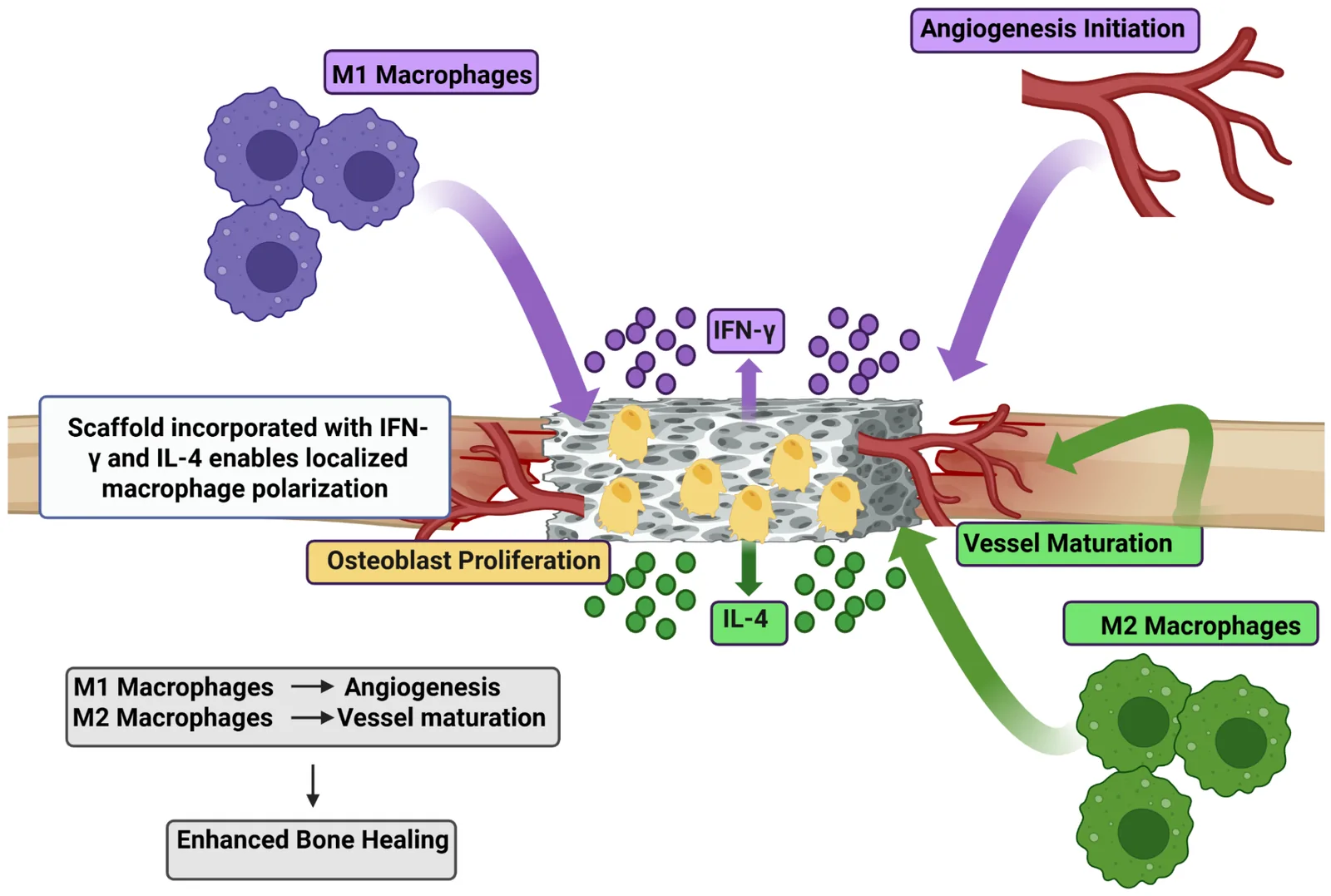
**Cytokine-releasing immunomodulatory scaffold regulating macrophage polarization for enhanced bone healing.** A cytokine-incorporated scaffold containing interferon-gamma (IFN-γ) and interleukin-4 (IL-4) locally induces macrophage polarization to coordinate vascular and osteogenic processes during bone repair. M1 macrophages initiate angiogenesis, whereas M2 macrophages promote vessel maturation and osteogenesis, collectively enhancing bone regeneration. Created in BioRender. Alexander, I. (2026) https://BioRender.com/xzufvy3 (Accessed on 20 July 2026).

Macrophage polarization into M1 (inflammatory) and M2 (anti-inflammatory) phenotypes represents a promising immunomodulatory strategy to enhance tissue repair following many injuries, including bone defects. Cytokines such as interferon gamma (IFN-γ) and interleukin (IL)-4 can be incorporated into scaffolds to induce M1 or M2 macrophage polarization, respectively (Figure 4) [86,87]. Localized cytokine delivery through scaffold systems offers a distinct advantage by directing macrophage activation at the injury site while minimizing systemic immune activation. In one experiment, researchers showed that silk scaffold materials incorporating cytokines showed potential to induce macrophage polarization [86]. Previous studies indicate that M1 macrophages initiate angiogenesis, while M2 macrophages promote vessel maturation, thereby enhancing the osteogenic process. Achieving this balance through cytokine-releasing scaffolds can significantly benefit bone regeneration [87]. Cytokines are also beneficial because they promote osteoblast proliferation, migration, and differentiation. However, several challenges exist with this approach. Despite these promising findings, successful clinical application depends on achieving precise temporal and spatial regulation of macrophage polarization. Improper macrophage activation may disrupt immune homeostasis through excessive pro-inflammatory signaling, whereas insufficient or inappropriate polarization may promote osteoclast formation and subsequent osteolysis, ultimately compromising bone regeneration [88]. Consequently, optimizing cytokine delivery and macrophage regulation remains essential for translating these immunomodulatory scaffold systems into effective therapies for osteomyelitis and bone repair.

Immunomodulatory effects have also been explored to assess the influence of macrophage polarization on the differentiation of osteogenic stem cells and bone restoration processes. Yang et al. demonstrated that scaffolds with larger pore sizes (400 µm) induced a greater M1-to-M2 macrophage transition than scaffolds with smaller pore sizes (0 µm and 200 µm). This shift promoted angiogenesis and the differentiation of osteogenic bone marrow mesenchymal stromal cells, suggesting that larger pore scaffolds may represent a promising strategy for polyetheretherketone-based bone tissue repair. These findings have since been supported to demonstrate that larger scaffold pore sizes promote macrophage phenotype transition and enhance the regenerative microenvironment [89,90]. Collectively, these findings suggest that scaffold architecture may play an important role in regulating the immune response during bone healing. Although these results are encouraging, further investigation is needed to optimize scaffold pore geometry and validate these immunomodulatory effects in clinically relevant models before widespread implementation [89,90,91].

### 3.7. Personalized and AI-Optimized Scaffold Design

**Figure 5:**
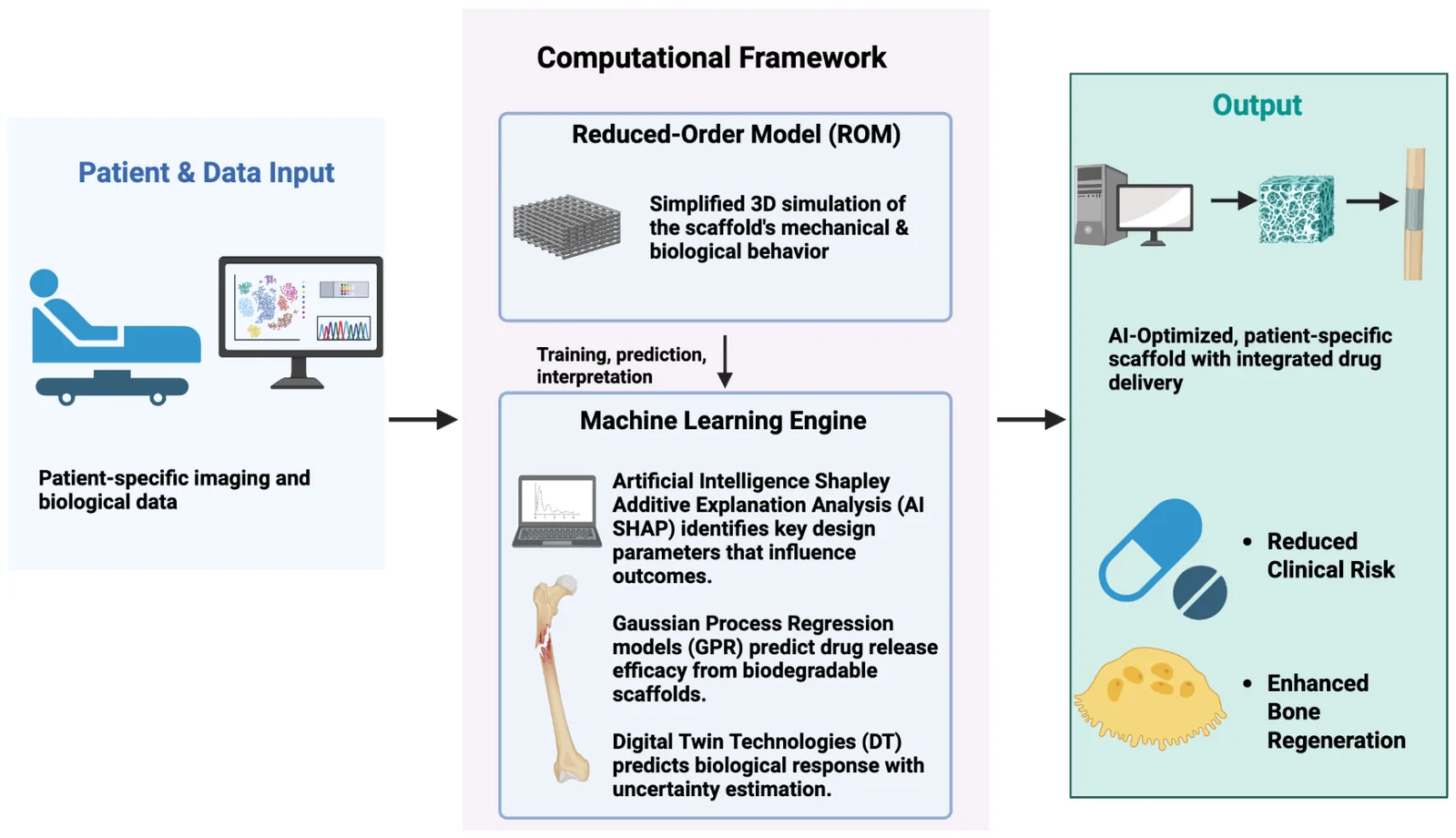
**Artificial intelligence-driven framework for personalized scaffold design in osteomyelitis treatment.** Artificial intelligence (AI) has been employed to design and optimize patient-specific bone scaffolds. The modeling approaches illustrated provide complementary tools for scaffold optimization, prediction of therapeutic performance, and the development of personalized treatment strategies. Created in BioRender. Lee, E. (2026) https://BioRender.com/wycexw4 (Accessed on 20 July 2026).

Drakoulas et al. presented a computational framework that integrates machine learning-based reduced-order models (ROMs), probabilistic modeling, and explainable artificial intelligence (AI) tools to optimize 3D-printed bone scaffold design (Figure 5). Within this framework, ROMs provide an efficient yet accurate computational representation of how bone scaffolds behave. These models are trained using probabilistic machine learning methods to provide prediction uncertainty and avoid overfitting. Additionally, they incorporate surrogate models to simplify complex, computationally intensive simulations, making optimization more efficient while accurately predicting outcomes such as bone regeneration [92]. Shapley Additive Explanation (SHAP) analysis is then used to understand which input parameters most influence outcomes, making the model transparent and interpretable. Together, these computational tools enable multi-objective optimization of scaffold design that, as these technologies continue to evolve, may facilitate customized scaffold properties, integrated drug delivery, reduced clinical risk, and assistance with surgical planning for osteomyelitis management [93]. Despite these promising capabilities, broader implementation of AI-guided scaffold optimization will require further refinement of current compilation models. Future frameworks should better account for dynamic tissue growth, chemical and nutrient diffusion, and the influence of electrical and other biological signaling pathways. Incorporating these complex, time-dependent variables will inevitably increase computational demands, reinforcing the importance of ROMs for maintaining efficient and clinically applicable scaffold optimization [26].

A Gaussian Process Regression (GPR) model was used to predict drug release efficacy from biodegradable scaffolds over time. This model captures patterns within the data while also estimating the uncertainty of its predictions [94]. It was trained and evaluated using experimental datasets to ensure reliable generalization across different scaffold formulations. This predictive framework was subsequently applied to drug-loaded electrospun nanofibers, which have shown significant promise as therapeutic scaffolds for applications ranging from cancer therapy to tissue regeneration. Through customizable fabrication methods and tunable fiber properties, electrospinning enables development of flexible fibrous materials optimized for controlled drug delivery [95]. Among the 28 machine learning models tested across eight model classes, including linear regression, decision trees, support vector machines, and neural networks, GPR demonstrated the best performance, achieving the lowest error rates and highest predictive accuracy for drug release. With an R^2^ of 0.931, the optimized GPR model explained over 93% of the variance in the experimental data, indicating strong predictive capability. These findings highlight the potential of machine learning approaches to optimize scaffold design and improve the prediction of drug release profiles. Despite this promising performance, broader implementation of GPR models remains limited by their relatively poor interpretability. The inability to fully explain how individual formulation parameters interact reduces mechanistic insight into drug release kinetics, underscoring the need for future models that balance predictive accuracy with biological interpretability [96].

Ahmed et al. proposed that, in the context of bone regeneration, digital twin (DT) technology offers a powerful tool for studying complex conditions like osteomyelitis by enabling real-time simulation, continuous monitoring, and prediction of biological responses to various treatment strategies. DTs provide valuable insights, dynamic data, and adaptive feedback to address the complexities of biofabrication and tissue engineering. By integrating data from multiple sources, such as imaging, sensors, and patient-specific information, DTs can facilitate the creation of personalized biofabricated tissues and organs [97]. They can also monitor the performance of biofabricated tissues over time and anticipate potential issues or complications, ultimately improving the safety and effectiveness of regenerative treatments [98]. Beyond these capabilities, DT technology has the potential to support personalized treatment planning by continuously adapting therapeutic strategies based on patient-specific biological responses. Despite this considerable potential, successful clinical implementation depends on the development of DT models that accurately capture the complexity and dynamic nature of biological systems. Achieving this goal will require advances in sensor technology, more sophisticated data analytics capable of managing highly variable biological datasets, and improved integration of machine learning algorithms to enhance model accuracy and predictive performance [24].

### 3.8. Bioinspired and Naturally Derived Antimicrobial Scaffolds

Silk-based biomaterials, particularly those derived from silk fibroin, offer a promising platform for musculoskeletal tissue regeneration, including bone, cartilage, ligaments, and intervertebral disks. These materials are biocompatible, biodegradable, mechanically robust, and can be molecularly engineered to mimic native tissue structures [99,100]. Beyond their favorable mechanical and biological properties, silk exhibits moderate intrinsic antibacterial activity while also enhancing the efficacy of antibacterial agents [101]. Silk-based scaffolds also support biomineralization essential for new bone formation and have demonstrated self-healing capabilities in preclinical studies, further supporting their potential for osteomyelitis treatment. Their compatibility with advanced fabrication techniques allows for patient-specific scaffold designs, and their smart material behavior enables adaptive healing responses. Despite these promising characteristics, the clinical translation of silk-based scaffolds remains dependent on further validation through well-designed clinical studies. As research continues to advance, silk fibroin-based scaffolds may provide a versatile platform for combining regenerative, antimicrobial, and patient-specific strategies within future osteomyelitis therapies [25].

Self-assembling peptide (SAP) hydrogels are promising biomaterials for bone tissue engineering, as they mimic the extracellular matrix and provide a foundation that supports osteogenic cell attachment, migration, proliferation, and differentiation [102]. These hydrogels form through non-covalent interactions such as hydrogen bonding, ionic interactions, and hydrophobic forces, creating nanofibrous networks capable of filling bone defects and promoting healing. Functionalization with specific peptide motifs, which are short sequences of amino acids designed to trigger desired cellular responses, can further enhance bone regeneration. Despite these favorable biological properties, the successful application of SAP hydrogels depends on achieving sufficient mechanical stability while preserving their regenerative capabilities. SAP hydrogels have limited mechanical strength and degrade rapidly, which can lead to premature cell release and inflammation, thereby restricting their use in load-bearing bone applications [103,104]. Future scaffold designs will likely focus on improving the mechanical durability of SAP hydrogels while maintaining the biological characteristics that make them attractive for bone tissue engineering.

### 3.9. Advanced Responsive Scaffolds for Targeted Bone Repair

**Figure 6:**
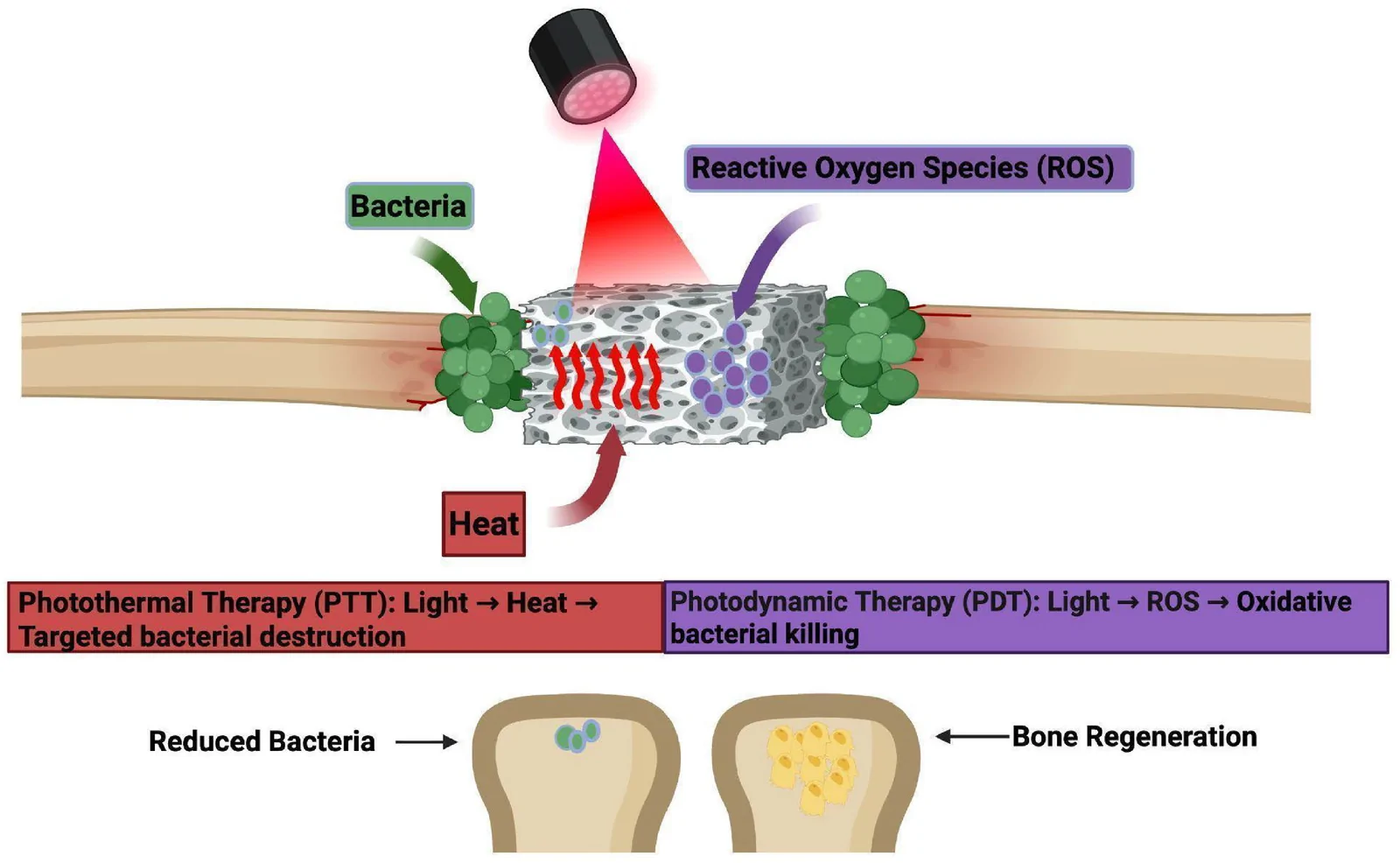
**Advanced responsive scaffolds for targeted bone repair.** Photothermal therapy (PTT) and photodynamic therapy (PDT) are emerging scaffold-compatible strategies for targeted osteomyelitis management that selectively eliminate bacteria within infected tissues. Together, these complementary approaches reduce bacterial burden while minimizing damage to surrounding healthy tissue. Created in BioRender. Lee, E. (2026) https://BioRender.com/pav3rvw (Accessed on 20 July 2026).

Wan et al. described phototherapy—including photothermal therapy (PTT) and photodynamic therapy (PDT)—as a non-invasive approach to treat osteomyelitis by selectively targeting infected tissue. PTT generates heat using light, while PDT produces reactive oxygen species to kill bacteria, both minimizing damage to healthy cells (Figure 6). Advances in nanotechnology have enabled multifunctional systems that combine these therapies with drug delivery and other treatments, improving overall effectiveness in infection control and bone regeneration [105]. Despite these therapeutic advantages, the clinical success of phototherapy depends on achieving adequate light penetration to the targeted treatment site. Optimizing light wavelength and delivery parameters is therefore essential to maximize therapeutic efficacy while minimizing attenuation within surrounding tissues [106]. Furthermore, understanding how different stages of bone healing influence light penetration is essential and will be critical for refining treatment protocols and improving clinical outcomes [107].

Gelmi et al. described stimuli-responsive biomaterials—activated by electricity, light, ultrasound, or magnetic fields—as promising platforms for guiding stem cell behavior and enhancing bone regeneration by promoting differentiation and increasing cellular activity within scaffolds. These materials can mimic or augment endogenous cues, such as dynamic tissue stiffness, electrical fields, mechanical strains, shear flow, and growth factors, which naturally regulate stem cell proliferation and differentiation [108]. By providing precise spatial and temporal control over these stimuli, scaffold systems can be engineered to optimize cellular responses and improve regenerative outcomes. Among these approaches, electrical stimulation delivered through conductive scaffolds is particularly promising, as many tissues naturally produce endogenous electrical signals that regulate angiogenesis, cell division, signaling, migration, and wound healing. Replicating these biophysical cues enables tissue engineering to more effectively direct stem cell fate [109]. Similarly, light-responsive systems allow precise spatial and temporal control over bioactive molecule release via light-triggered reactions. Together, these stimuli-responsive strategies offer versatile approaches for modulating stem cell migration, proliferation, differentiation, and extracellular matrix deposition to enhance bone regeneration. Despite their considerable therapeutic potential, successful clinical translation depends on overcoming several modality-specific challenges. Conductive scaffolds require careful control of electrical stimulation parameters, as variations in signal strength and duration can significantly influence cellular behavior and may limit their reproducibility and clinical translation [110]. Likewise, the clinical applicability of light-responsive scaffolds remains inherently limited by the restricted penetration of light through biological tissues, reducing their effectiveness in targeting deeper regions [63]. Future research should therefore focus on optimizing stimulus delivery while improving the reproducibility and clinical applicability of these multifunctional scaffold systems.

### 3.10. Next-Generation Bioinks and 3D Bioprinting Strategies

Drakoulas et al. highlighted recent studies investigating both computational and experimental approaches to develop 3D-printed, biocompatible scaffolds for severe bone fractures. These scaffolds mimic natural bone by providing a porous, three-dimensional framework that supports cell attachment, growth, and differentiation [111,112,113]. Scaffold designs generally fall into two categories: (1) lattice-based structures, which optimize strength and nutrient flow, and (2) biomimetic structures, which replicate bone architecture to better support cellular responses. Each design offers distinct mechanical and biological advantages; however, there is no consensus on which architecture provides the greatest overall therapeutic benefit. Consequently, computational models remain valuable for predicting outcomes and guiding experimental validation before clinical implementation. Although these scaffold designs have not yet been extensively evaluated for osteomyelitis, they represent promising platforms whose successful translation will depend on continued in vitro and in vivo validation to identify the most effective scaffold architecture for clinical use [26].

Li et al. described shape memory polymers (SMPs) as an emerging class of biomaterials in bone tissue engineering due to their unique functional properties. SMPs are typically composed of polymers such as polycaprolactone, polyurethane, polylactic acid, and poly(L-lactide), which are immersed in molten salt or sugar, cured, and undergo additional processing before being stabilized through physical or chemical crosslinking. They are valued for their biocompatibility, biodegradability, favorable mechanical properties, and minimally invasive application [114]. Their ability to change shape, known as the shape memory effect (SME), makes them particularly useful for irregular bone defects [115]. This effect relies on two phases: a fixed phase that maintains the original shape, and a reversible phase that holds a temporary shape until triggered by stimuli such as heat, water, magnetic fields, or ultrasound. Upon activation, SMPs can expand to fit bone defects precisely, creating a tight interface that supports bone growth. Studies have shown that combining shape memory capabilities with biodegradability holds significant promise for advancing tissue engineering. Despite these promising characteristics, the successful application of SMPs in bone regeneration depends on overcoming important mechanical and translational challenges. SMPs still face limitations in repairing complex or load-bearing bone defects due to the demanding mechanical and structural requirements of bone tissue. Furthermore, their potential has been demonstrated primarily in cell-based studies and animal models, particularly for cranio-maxillofacial defects, with limited investigation in limb bone repair and no reported clinical studies in humans [116]. Future research should therefore focus on validating the safety, mechanical performance, and clinical efficacy of SMP-based scaffolds in human studies before they can be considered viable alternatives for widespread clinical application [114].

### 3.11. Clinical Translation and Current Clinical Evidence

Although the scaffold strategies reviewed in this article encompass a broad range of therapeutic mechanisms, they differ substantially in their stage of clinical translation, with only antibiotic-loaded calcium sulfate carriers and bioactive glass currently being used in routine clinical practice. These materials are commercially available and in routine use, including gentamicin- or vancomycin-loaded calcium sulfate/hydroxyapatite (CERAMENT G/V), point-of-care antibiotic-loaded calcium sulfate (Stimulan), and bioactive glass S53P4 (BonAlive). A systematic review and meta-analysis of antibiotic-loaded calcium sulfate in chronic osteomyelitis reported an overall infection eradication rate of 92%, although delayed wound healing occurred in 20% of patients and aseptic wound leakage in 12%, and the eradication rate did not differ significantly between tobramycin-loaded and vancomycin plus gentamicin-loaded formulations [117]. Additional clinical support comes from the only placebo-controlled randomized trial in this field, in which all post-surgical infective complications in diabetic foot osteomyelitis occurred in patients receiving unloaded calcium sulphate granules and none in those receiving antibiotic-impregnated granules, although the trial enrolled only twenty patients at a single center and was terminated early at interim analysis [118].

In addition to antibiotic-loaded calcium sulfate, bioactive glass represents one of the few scaffold technologies that has demonstrated successful translation into routine clinical practice. Bioactive glass S53P4 represents a distinct approach, achieving intrinsic antibacterial activity through local ion release and pH elevation without an incorporated antibiotic, thereby avoiding the resistance concerns inherent to antibiotic-eluting carriers. A meta-analysis of studies reporting outcomes at twelve months or longer found a pooled infection eradication rate of 88.1% across chronic long bone, diabetic foot, and other osteomyelitis etiologies [119]. Similarly, a direct comparison in cavitary chronic osteomyelitis found no significant difference in infection recurrence or complication rates between bioactive glass and antibiotic-loaded calcium sulphate beads [120].

By contrast, the antimicrobial peptide, CRISPR-based, quorum sensing, nanoparticle, immunomodulatory, stimuli-responsive, bioinspired, and computationally optimized platforms described in this review remain largely confined to in vitro and preclinical investigation, with little or no available human clinical evidence. Notably, the clinical evidence supporting even these established materials consists almost entirely of observational cohorts and case series rather than randomized trials, indicating that one of the principal barriers to translation is not material performance alone but the scarcity of adequately powered randomized evidence. A comprehensive comparison of the scaffold strategies discussed throughout this review, including their therapeutic mechanisms, primary advantages, current clinical translation, and translational challenges, is provided in Table 1. Future clinical translation of these emerging scaffold technologies will depend not only on continued advances in biomaterial design but also on the generation of high-quality clinical evidence demonstrating their safety, efficacy, and long-term therapeutic benefit.

## 4. Discussion

Osteomyelitis remains a significant clinical challenge due to persistent infection and high recurrence rates despite current gold-standard treatments. Approaches such as the Masquelet technique, which utilizes antibiotic-loaded PMMA cement spacers, have improved the management of bone defects following debridement. Nevertheless, important limitations remain in achieving both reliable infection eradication and sustained bone regeneration. Emerging scaffold-based biomaterials—including antimicrobial-loaded matrices, bioactive materials, and smart, stimuli-responsive systems—offer promising strategies to address these challenges. By integrating localized antimicrobial delivery with structural and regenerative support, these technologies have the potential to enhance drug delivery efficiency, improve tissue integration, and promote more effective bone healing in the treatment of osteomyelitis.

The successful application of these scaffolds, however, depends on several critical biological and mechanical considerations. Mechanical strength and degradation rates must be carefully optimized as many bioactive ceramics and polymer-based scaffolds exhibit brittleness or insufficient load-bearing capacity in osteomyelitic bone. Furthermore, scaffold architecture, pore size, and material composition play central roles in regulating vascularization and macrophage-mediated immune modulation, both of which are essential for infection clearance and bone regeneration. Safety considerations also remain important as excessive concentrations of metallic ions or toxic polymer degradation products may impair osteogenesis or provoke adverse inflammatory responses.

Beyond material design, translational and technological challenges continue to limit clinical adoption of advanced scaffold systems. Personalized patient-specific scaffolds generated via 3D bioprinting, AI-guided optimization, or digital twin technologies offer promising solutions for complex and irregular bone defects. However, these approaches rely on high-quality patient data, robust predictive modeling, and experimental clinical validation. Additionally, scaling multifunctional scaffolds that combine controlled drug release, antimicrobial activity, and bioactivity while meeting manufacturing standards remains a major hurdle for clinical translation.

Despite these challenges, continued advances in biomaterial engineering, targeted drug delivery systems, immune-modulatory strategies, and computational design approaches hold substantial promise for improving osteomyelitis treatment. The integration of these technologies may ultimately enable the development of safer, more effective, and personalized scaffold-based therapies capable of simultaneously addressing infection and bone regeneration.

## 5. Conclusions

Future research in osteomyelitis treatment should focus on integrating advanced biomaterials, immunomodulation, and precision medicine approaches to enhance scaffold efficacy and clinical applicability. Looking ahead, the greatest advances will likely arise from multifunctional scaffold platforms that integrate personalized scaffold design with the stimuli-responsive drug delivery, immunomodulation, antimicrobial therapies, and AI-guided optimization within a single therapeutic construct. Such integrated systems have the potential to deliver patient-specific therapies that simultaneously improve infection control, bone regeneration, and overall treatment outcomes.

In parallel, smart and stimuli-responsive scaffolds that respond to local environmental cues such as pH, temperature, or electrical signals could provide dynamic support for bone regeneration while minimizing systemic side effects. Continued development of bioactive and bioinspired materials, including silk fibroin, self-assembling peptide hydrogels, and shape memory polymers, may further enhance both infection control and tissue repair. Clinical translation will require standardized manufacturing methods, scalable production, regulatory approval pathways, and cost-effective implementation before these technologies can be routinely incorporated into osteomyelitis management. By simultaneously addressing infection eradication and bone regeneration through personalized, multifunctional scaffold systems, emerging tissue-engineered therapies have the potential to transform the future management of osteomyelitis.

## Figures and Tables

**Table 1:** **Comparative Overview of Biomaterial Scaffold Strategies, Therapeutic Mechanisms, and Translational Challenges in Osteomyelitis Management**. This table compares the biomaterial scaffold strategies discussed in this review, highlighting their core concepts and materials, therapeutic mechanisms, and major translational challenges in osteomyelitis management.

Section	Core Concepts & Materials	Primary Advantages	Key Challenges & Limitations	References
Section 3.1. Biomaterial-Based Scaffold Strategies for Osteomyelitis Treatment	- 3D porous biomaterials & Extracellular matrix (ECM)	- Prevents biofilm formation while regenerating bone. - Delivers antibiotics with tailored kinetics and antimicrobial ions. - Osteoconductive: Guides cell migration. - Osteoinductive: Promotes cell differentiation and maturation of osteogenic cells.	- True osteogenesis strictly requires the presence and activity of viable, active bone-forming cells, not just the material framework alone.	[24,25,26,27,28,29]
Section 3.2. Antimicrobial-Loaded Scaffolds	- Antibiotic coatings - Antimicrobial Peptides (AMPs) - Metallic ions (Silver)	- Establishes a local aseptic environment. - AMPs disrupt bacterial membranes and neutralize endotoxins (*S. aureus*, *E. coli*). - Silver ions disrupt microbial DNA, inhibit biofilms, and offer a safe therapeutic window (10–33 μM). - Antibiotic-loaded calcium sulfate carriers are currently used in routine clinical practice.	- Residual bacteria can evade surgical debridement and develop resistance. - AMPs suffer from poor in vivo stability, high production costs, and dose-dependent cytotoxicity. - Metals risk long-term systemic toxicity (organ dysfunction, oxidative stress).	[30,31,32,33,34,35,36,37,38,39,40,41,42,43,44,45]
Section 3.3. Bioactive, Osteoconductive, & Osteoinductive Scaffolds	- Hydroxyapatite (HA) - Calcium Phosphates (CaPs: α-TCP, β-TCP) - Bioglass - Polymers (Chitosan/Gelatin composites)	- CaPs mimic bone’s inorganic phase (70%) to bond directly with bone and boost cell proliferation. - Bioglass raises local pH for antibacterial action and releases ions to stimulate osteoblastic genes. - Composite blends allow for mechanical advantage and integration of favorable biological properties. - Bioactive glass (S53P4) is currently used in routine clinical practice.	- HA and bioactive ceramics are inherently brittle and prone to structural damage when used alone. - Pure chitosan lacks the mechanical strength required for load-bearing bone repairs.	[27,46,47,48,49,50,51,52,53,54,55,56]
Section 3.4. Smart and Responsive Scaffolds	-Stimuli-responsive 3D hydrogel networks (pH, temperature, light, magnetic fields)	- Provides highly targeted, on-demand drug delivery triggered by local environmental shifts (e.g., infection-induced fever or pH drops). -High water content protects encapsulated drugs, minimizes systemic side effects, and reduces dosing frequency.	- Hydrogels can suffer from delayed or insufficient localized responsiveness. - Notable structural vulnerabilities, including poor mechanical strength, loading/handling difficulties, and a distinct lack of clinical trial validation.	[58,59,60,61,62,63,64,65,66,67]
Section 3.5. Advanced Antimicrobial Strategies in and Adjacent to Scaffolds	- CRISPR-Cas9 phages - Quorum Sensing (QS) inhibitors - Hybrid drug/ion delivery - Nanoparticles (MSNs, PLGA, silver)	- CRISPR: Sequence-specific pathogen DNA cleavage (resolves soft tissue infections, resensitizes strains). - QS Inhibitors: Interferes with auto-inducing signaling molecules and receptors to halt biofilm creation. - MSNs: High surface area and bioactivity maximize cellular adhesion and payload delivery.	- CRISPR phages fail to fully clear deep bone infections alone, necessitating combination with surgical debridement. - QS inhibitors suffer from molecular instability, cytotoxicity, and unknown impacts on friendly bacteria. - PLGA polymers generate acidic, inflammatory degradation byproducts.	[68,69,70,71,72,73,74,75,76,77,78,79,80,81,82,83,84,85]
Section 3.6. Immunomodulatory Scaffolds for Infection Control	- Cytokine-loaded matrices (IFN-γ and IL-4) - Large-pore engineered materials (400 µm) - Silk and polyetheretherketone (PEEK) bases	- Activates target immune cells locally rather than systemically. - M1 macrophages initiate angiogenesis, while M2 macrophages promote vessel maturation. - Cytokines drive osteoblast growth and migration. - Larger pore sizes (400 µm) inherently accelerate the critical M1-to-M2 phenotype transition to enhance stem cell repair.	- Improper or poorly timed macrophage activation can disrupt immune homeostasis via dangerous pro-inflammatory cascades. - Faulty activation can inadvertently stimulate osteoclasts, causing bone destruction (osteolysis). - This is an early-stage, newly explored technique that demands significant future validation.	[86,87,88,89,90,91]
Section 3.7. Personalized & AI-Optimized Scaffold Design	- Machine Learning Reduced-Order Models (ROMs) - Shapley Additive Explanation (SHAP) interpretability analysis - Gaussian Process Regression (GPR) - Digital Twin (DT) technology	- ROMs and SHAP optimize 3D scaffold design efficiently with clear input transparency. - GPR predicts drug release kinetics from electrospun nanofibers with high accuracy (R^2^ = 0.931). - DTs integrate real-time patient data and sensor feedback to monitor tissue performance and predict complications.	- GPR is limited by its poor interoperability - ROMs and SHAP struggle to simulate complex, time-dependent factors like dynamic tissue growth and nutrient diffusion. - DTs are bottlenecked by the need for more advanced sensors and data analytics.	[24,25,92,93,94,95,96,97,98]
Section 3.8. Bioinspired and Naturally Derived Antimicrobial Scaffolds	- Silk fibroin biomaterials - Self-Assembling Peptide (SAP) hydrogels - Electrically conductive scaffolds	- Silk is mechanically robust, supports biomineralization, and possesses mild intrinsic antibacterial traits. - SAPs form nanofibrous networks mimicking the natural ECM. - Conductive materials replicate natural electrical fields to direct stem cell healing.	- SAP hydrogels degrade too rapidly and lack the mechanical strength for load-bearing applications. - Conductive frameworks require hyper-precise electrical parameter controls to ensure reproducible cell behavior.	[25,99,100,101,102,103,104]
Section 3.9. Advanced Responsive Scaffolds for Targeted Bone Repair	- Photothermal (PTT) & Photodynamic (PDT) therapies - Conductive & light-responsive scaffolds - Multi-stimuli frameworks (ultrasound, magnetic fields)	- PTT (heat) and PDT (reactive oxygen species) selectively target pathogens non-invasively with minimal healthy cell damage. - Replicates natural biophysical/electrical signals to direct stem cell migration, division, and blood vessel formation. - Light activation allows precise spatial/temporal control over drug release.	- Phototherapy efficacy is highly restricted by biological tissue scattering, preventing light from reaching deeper scaffold regions. - Electrical stimulus relies on hyper-precise parameter tuning (duration/signal strength); slight variations disrupt cell behavior, limiting clinical translation and reproducibility.	[63,105,106,107,108,109,110]
Section 3.10. Next-Generation Bioinks & 3D Bioprinting Strategies	- 3D-printed architectures (Lattice vs. Biomimetic structures) - Shape Memory Polymers (SMPs)	- Lattice models optimize strength/nutrient flow; biomimetic designs replicate native bone architecture. - SMPs use a dual fixed/reversible phase triggered by external stimuli (heat, water, ultrasound) to expand and seamlessly fit irregular bone defects. - Supports minimally invasive delivery methods.	- There is no established structural consensus on the best 3D print design, and true efficacy in osteomyelitis has not yet been demonstrated. - SMPs lack the strict mechanical integrity required for heavy, load-bearing bone segments (e.g., limbs). - Data is restricted to cell models and animal cranio-maxillofacial defects; there are zero human clinical applications to date.	[111,112,113,114,115,116]

## Data Availability

No new data were created or analyzed in this study. Data sharing is not applicable to this article.

## Abbreviations

The following abbreviations are used in this manuscript:

*S. aureus*	*Staphylococcus aureus*
*E. coli*	*Escherichia coli*
PMMA	Polymethyl methacrylate
AMP	Antimicrobial peptide
LPS	Lipopolysaccharide
MRSA	Methicillin-resistant *Staphylococcus aureus*
RGD	Arginine-glycine-aspartic acid
HA	Hydroxyapatite
CaP	Calcium phosphate
ɑ-TCP	Alpha-tricalcium phosphate
ꞵ-TCP	Beta-tricalcium phosphate
CRISPR	Clustered regularly interspaced short palindromic repeats
sgRNA	Single guide RNA
CRISPR RNA	crRNA
tracrRNA	Trans-activating crRNA
QS	Quorum signaling
NPs	Nanoparticles
MSN	Mesoporous silica
PLGA	Poly(lactic-co-glycolic) acid
IFN-γ	Interferon-gamma
IL-4	Interleukin-4
ROMs	Reduced-order models
AI	Artificial intelligence
SHAP	Shapley additive explanation
GPR	Gaussian process regression
DT	Digital twin
SAP	Self-assembling peptide
PTT	Photothermal therapy
PDT	Photodynamic therapy
ROS	Reactive oxygen species
SMPs	Shape memory polymers
SME	Shape memory effect
